# Evaluation of the danger of a tailings pile belonging to an active mine through its characterization and a dispersion model

**DOI:** 10.1007/s10661-023-11475-4

**Published:** 2023-06-26

**Authors:** Jesús Fidel González-Sánchez, Osiel O. Mendoza-Lara, Jorge Luis Romero-Hernández, Georgina Fernández-Villagómez

**Affiliations:** Faculty of Engineering, National Autonomous University of Mexico, Av. Universidad 3000, C.P. 04510 Del. Coyoacán, Ciudad de Mexico, México

**Keywords:** Air pollution, Modelling, Mining tailing, AERMOD, WRF

## Abstract

Mining is one of the principal economic activities in Mexico, which in addition to bringing benefits to the population, causes health and environmental problems. This activity produces a lot of wastes, but the main one is tailings. In Mexico, these wastes are disposed of in the open air, and there is no control over them, so the particles of these wastes are dispersed by wind currents to the surrounding population. In this research, tailings were characterized, finding in them particles smaller than 100 microns; in this way, tailings can enter into the respiratory system and hence can cause diseases. Furthermore, it is important to identify the toxic components. The present work does not have previous research in Mexico, and it shows a qualitative characterization of the tailings from an active mine using different analytical techniques. In addition to the data obtained from the characterization of the tailings, as well as the concentration of the toxic elements found, which were Pb and As, a dispersal model was generated and used to estimate the concentration of particles in the wind generated at the studied area. The air quality model used in this research is AERMOD, where it uses emission factors and available databases provided by Environmental Protection Agency (USEPA); Moreover, the model was coupled with meteorological information from the latest generation WRF model. The modeling results estimated that the dispersion of particles from the tailings dam can contribute up to 10.15 µg/m^3^ of PM_10_ to the air quality of the site, which, according to the characterization of the samples obtained, could be dangerous for human health and can be estimated up to a concentration of 0.04 µg/m^3^ of Pb and 10.90 ng/m^3^ of As. It is very important to make this kind of research to know the risk which people around this disposal sites are exposed to.

**Supplementary Information:**

The online version contains supplementary material available at 10.1007/s10661-023-11475-4.

## Introduction

Nowadays, the necessity to manufacture products to satisfy human needs, whose essential elements are metals, has increased (Armienta et al., 2016; Xiaolong et al., 2021). In Mexico, mining has belonged to the main economic activities since colonial times and has currently contributed significantly to economic development. Mainly extracting: silver, gold, copper, zinc, and lead. However, this activity affects the environment and generates considerable amounts of waste. Mine tailings (or “jales”, as they are known in Mexico), refer to the materials that are left over after the valuable substances have been removed at the end of mining or mineral processing. They are the primary residues produced in the metal extraction process, they contain significant concentrations of potentially toxic metals and metalloids (Corrales-Pérez & Romero, 2013; Dótor-Almazán et al., 2017; Zúñiga-Vázquez et al., 2018). Tailings may contain Ni, Cu, Fe, Zn, and Pb, in relatively high concentrations (0.5 to 3%) and sometimes Ag and Au. However, these residues can also contain potentially toxic elements, such as arsenic, in concentrations of up to 100 mg/kg. (Ceniceros-Gómez et al., 2018; Falagán et al., 2017).

Historically, tailings were frequently placed in convenient areas, occasionally even in rivers or drainage systems (Ritcey, 2005; Vick, 1990). Currently, a range of approaches has been devised to handle mining waste. These approaches encompass the placement of dehydrated or thickened tailings in self-supporting deposits or stockpiles, their application as fill material in underground and open-pit mines, subaqueous disposal, and the prevailing method, which entails depositing tailings in open air dams (Carrillo López et al., 2016). In Mexico, mine tailings deposits principally are carried out in piles, depending on the type of mineral and the applicable environmental regulation. However, it is important to note that the construction of new tailings dams is currently regulated and restricted due to the environmental risks they pose (SEMARNAT, 2003). Managing mine tailings is not easy and represents trouble because the piles that contain them can have problems from the dispersion of material, have structural failures, cause spills and generate acid drainage the disposal is done in open-air conditions (Hudson-Edwards, 2016; Kossoff et al., 2014; Loredo-Portales et al., 2020). Sometimes these mining piles are abandoned and the dispersion of tailings is favored, either by hydraulic or wind processes (Loredo-Portales et al., 2020; Sánchez-Donoso et al., 2019).

One of the leading environmental problems in Mexico is the lousy management of tailings due to mining being one of the principal economic activities since colonial time (García et al., 2017; Meza-Figueroa et al., 2009; Suter, 2016). During the windy season, the dispersion of the particles in the environment can be seen with the naked eye. According to literature, it is known that mining tailings are very fine-grained. The consist of particles ranging from sizes of 1–600 μm; so they can be classified according to aerodynamic diameter: PM_2.5_ and PM_10_ (less than 2.5 µm and less than 10 µm respectively), based on their respective health impacts (Ceniceros-Gómez et al., 2018; World Health Organization, 2013).

Carrying out an impact assessment on the surrounding areas of a mining site, as well as the surrounding ecosystems and human settlements, it is complicated since assessing of risks to human health is needed. (Corrales-Pérez & Romero, 2013; Kossoff et al., 2014; Solà et al., 2004). However, a first approximation of the site’s dangerousness could be made based on the characterization of the particulate matter from tailings using different analytical techniques and determining the total concentrations of potentially toxic elements and comparing them with the maximum permissible limits established by governmental or international organizations (Gavilán-García et al., 2020; Loredo-Portales et al., 2020; Salas Urviola et al., 2020a, 2020b). Dispersion modeling of tailings’ particles into the air can be done by having the availability of data and representative inventories of particle emissions that are essential for an environmental risk assessment and provide the basis for the analysis of the environmental fate of these tailings’ emissions (Richardson et al., 2019a, 2019b). For this reason, to estimate the emission factors, the following equation can be used (“Emission Factors for Air Pollutants Related to Mining and Mineral Processing,” 2016).

$${\mathrm{PME}}_{\mathrm{process}} = \left({\mathrm{PMEF}}_{\mathrm{process}}\right)\left({\mathrm{Unit}}_{\mathrm{process}}\right)\left(1-\frac{\mathrm{EC}}{100}\right)$$

where

$${\mathrm{PME}}_{\mathrm{process}} =$$
particulate matter emissions for a given process, lbs or kg.
$${\mathrm{PMEF}}_{\mathrm{process}} =$$
particulate matter emission factor for a given process; lbs unit^−1^ or kg unit.^−1^
$${\mathrm{Unit}}_{\mathrm{process}} =$$
tons (processed, produced, charged, transferred), miles traveled, kg solvent used, etc.
$$\mathrm{EC}$$
= emission control factor, %

This work aims to evaluate the danger of tailings belonging to an active mine in Mexico through its tailings characterization and dispersion modelling of particles present in them, so that in subsequent studies, a treatment of these residues can be proposed to mitigate environmental and health damage. With the information obtained from this research, it is intended to be beneficial to the environmental regulatory entity of Mexico, the academy and the environment.

## Materials and methods

### Samples

Ten samples of mining wastes were obtained following the NMX-AA-132-SCFI-2006 procedure (Secretaría de Economía, 2006). The sampling was superficial in an area of approximately 15 ha. Samples were taken at a depth of 10 cm, and approximately 5 kg of wastes were collected and stored in plastic bags (Figure S1, Supplementary Materials).

### Characterization of samples

#### pH and humidity

The pH was determined at each point using the Corning pH-30 Sensor equipment, and deionized water was used throughout. The collected samples were then transferred in polyethylene bags and stored at 4 °C in a cold room of the laboratory of Sanitary and Environment Engineering of Engineering Faculty of the UNAM. The humidity was later determined following the European standard UNE-EN ISO 17892–1 (BS EN ISO 17892–1:2014, 2004).

#### Size of the particles and chemical characterization

Afterward, physical properties of the samples were determined: granulometry using the UNE-EN 933–1 standard (BS EN 933–1:2012, 2012) and particle size using the Philips XL20 scanning electron microscope to 20 kV of power with a 4-mm spot and BSE detectors (electro scattered electrons) located in the materials laboratory of the Mechanical and Electrical Engineering Division (DIMEI) of the Faculty of Engineering, UNAM. For the chemical and structural properties, a punctual elemental analysis was carried out using an Oxford microprobe with 10 mm glass and Inca SL-20 software which is in the aforementioned laboratory. The composition of samples was determined employing the Infrared technique (FTIR), the analysis was carried out in Laboratory of Environmental Molecular Geochemistry of Institute of Geology, UNAM with a Thermo Scientific Nicolet iS10 brand FTIR spectrometer using a GladiATR accessory with a diamond crystal under the entire mid-infrared range (400–4000 cm^−1^). The reading of the samples consisted of 64 scans with a resolution of 4 cm^−1^, applying a background at the beginning of each analysis. All the samples were analyzed in nitrogen (NUAP type) to ensure the absence of moisture and CO_2_.

#### Elemental characterization

To determine the total lead and arsenic concentration, digestion of the samples was carried out using a CEM microwave unit, model Mars 6.0. Ten milliliters of aqua regia (25% HNO_3_, Baker, 70% and 75% HCl, Baker, 36.5–38%) were added along with 0.5 g of sample in each tube of the microwave. Then, for 20 min the temperature was increased until it reached 200 °C and was held for another 15 min. Samples were then left to cool for 10 min and filtered with a nylon membrane with a pore size of 45 microns.

As and Pb were determined in digests using a GBC AVANTA atomic absorption spectrophotometer equipped with a GBC HG 3000 peristaltic pump. The concentrations in digests and extracts were determined using Hydride Generation-Atomic Absorption Spectroscopy for As and Flame Atomic Absorption Spectroscopy for Pb, following the methodology described in literature (Environmental Protection Agency, 1994, 2007; García et al., 2017). For all the solutions de-ionized water 18 MΩ cm, Millipore Milli-Q system were used. Moreover, two calibration curves were prepared, one for As and one for Pb with a concentration range of 5 to 50 ng/L and 5 to 30 mg/L respectively. All samples were analyzed by triplicate.

### Air dispersion model

A dispersion air quality model was used to evaluate the impact of the mining tailings in the zone. The primary data in air quality systems are information of topography, meteorology, and pollutant emissions (González-Rocha et al., 2015). In the following section, these variables are described. To predict and model airborne concentrations of suspended particulate matter, AERMOD has been the dispersion model with promising results (Tartakovsky et al., 2013).

#### Wind direction

Due to the scarce meteorological information in the study area, it was decided to obtain the data through a meteorological model. The data obtained was input for the Weather Research and Forecasting (WRF) model. The results identified in the direction and speed of the wind (Figure S2, Supplementary Materials), showing the wind rose representative of the study area. The dominant wind direction comes from the northeast, with 31.5% of the frequency with maximum speeds of 11 m/s. According to the modeled database, the average wind speed is 3.69 m/s, and the calms are below 1.91% of the data.

#### AERMOD

AERMOD View (paid software) version 10 with the model code for AERMOD Version 22,112 (US EPA). The supplier company is Lakes Software. AERMOD is an air quality modeling system developed in the USA by the AMS and the EPA (American Meteorological Society and the Environmental Protection Agency EPA, respectively) (Kumar et al., 2016). The Gaussian dispersion model (Tran et al., 2019) contains building downwash algorithms and advanced meteorological calculations(Kalhor & Bajoghli, 2017). One of the most important characteristics of the model is that it can execute with real or estimated meteorological information (USEPA, 2018).

Additionally, the model can estimate the concentration of air pollutants: (1) the outputs of the model are 1-h average concentrations (Pandey & Sharan, 2019), (2) and the recommendation that it applies to study areas less than 50 km from the source (Mokhtar et al., 2014). The software requires meteorological and terrain inputs through two preprocessors, AERMET and AERMAP.

Regarding meteorology, the modeling system considers variables such as temperature, dew point, pressure, solar radiation (O’Shaughnessy & Altmaier, 2011), wind speed, wind direction, total and low cloud cover, convective velocity scale, temperature scale, mixing height, and surface heat flux (Ma et al., 2013). The preprocessor results are directly introduced to AERMOD (Huang & Guo, 2019). AERMOD is compatible with many weather models which can ease predicting the particle dispersion (Seangkiatiyuth et al., 2011).

Given that the terrain is complex in the area of study, AERMOD calculates the concentrations and simulates a plume as a sum horizontally and vertically, taking into account the different elevations in the area (Zou et al., 2010). The database with the best resolution was obtained from the U.S. Geological Survey (USGS), while the digital model used was the Shuttle Radar Topography Mission (SRTM) with a resolution of 30 m.

In the case of the receptors used for calculating concentrations, a 50 × 50-km grid with 0.5 km of spacing (de Ferreyro Monticelli et al., 2020) generating a total of 10,201 receptors is considered to estimate the airborne pollutants in the area. Having as a reference point the following UTM coordinate (Zone 14): 459,922.05 m N, 2,292,568.0 m E.

Surface and vertical weather data can be obtained from meteorological stations near the study area. However, in Mexico and other countries, there are regions where it takes more work to install and operate weather stations to obtain surface and vertical weather information. Therefore, it is essential to generate databases, protocols, guides, standards, and indexes to improve the accuracy of models and the diagnosis that would enhance air quality management (Mendoza-Lara et al., 2021).

In the present work, weather stations are distant, and their data may not be representative. Therefore, WRF was used, a state-of-the-art mesoscale numerical weather prediction model designed for both atmospheric research and operational forecasting applications. This model helps attach weather parameters required for air quality models (Kesarkar et al., 2007).

WRF modeling results were coupled using Mesoscale Model Interface (MMIF) developed by USEPA. Table 1 shows the parameters used in weather modeling.

**Table 1:** Weather modeling parameters

Element	Parameter
Forecasting modeling system	WRF
Resolution	12 km
Study area	50 km × 50 km
Period	365 days (1st of January to 31st of December) of 2018
Database	GRIB2 ds083.2 NCEP Climate Forecast System Version 2
Reference coordinates	459,922.05 m N, 2,292,568.0 m E., Zone 14

## Results and discussions

### Mining tailings characterization

#### pH and humidity

The pH and humidity are shown in Figure S3 and S4, Supplementary Materials. The samples have a slightly neutral-alkaline pH, this may be due to the fact that there are minerals with neutralization capacity such as carbonates and a low concentration of acid-forming components such as pyrites (Armienta et al., 2004; Corona Sánchez et al., 2021; Espinosa et al., 2009). Due to these values, it can be said that there is also a low mobility towards aquifers of dangerous toxic elements because it is necessary to have acid conditions in the environment for this to happen (Salas-Luevano et al., 2021; Salas Urviola et al., 2020a, 2020b). It should be noted that the survey was carried out from the top to the base of the dam (Figure S1), beginning with point z-1 and as the numbering increases, it is closer to the base with point z-10. The average humidity of the site is 8% and there is greater humidity at point z-3 because it is the point of deposit of new tailings, and they come out in the form of sludge. However, over time the tailings dry up due to the environmental conditions of the site and become a loose, dry, and sandy form that is very easy to disperse in the air as it is the case at points of z-6 to z-8 with the lowest humidity, where at first sight the tailings were found in that way (Figure S5, Supplementary Materials).

#### Size of the particles and chemical characterization

**Fig. 1:**
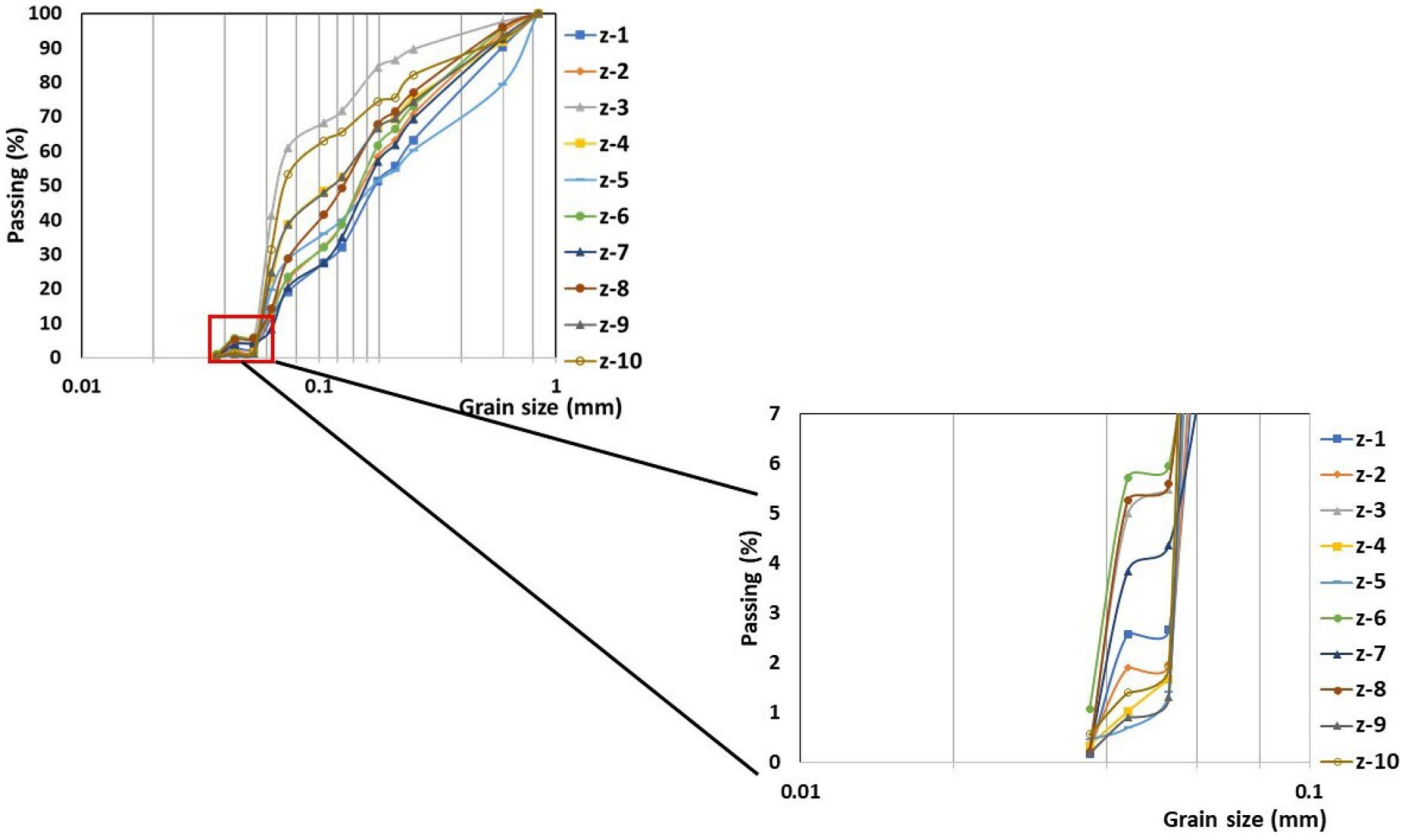
Granulometry of the different sampling points

**Fig. 2:**
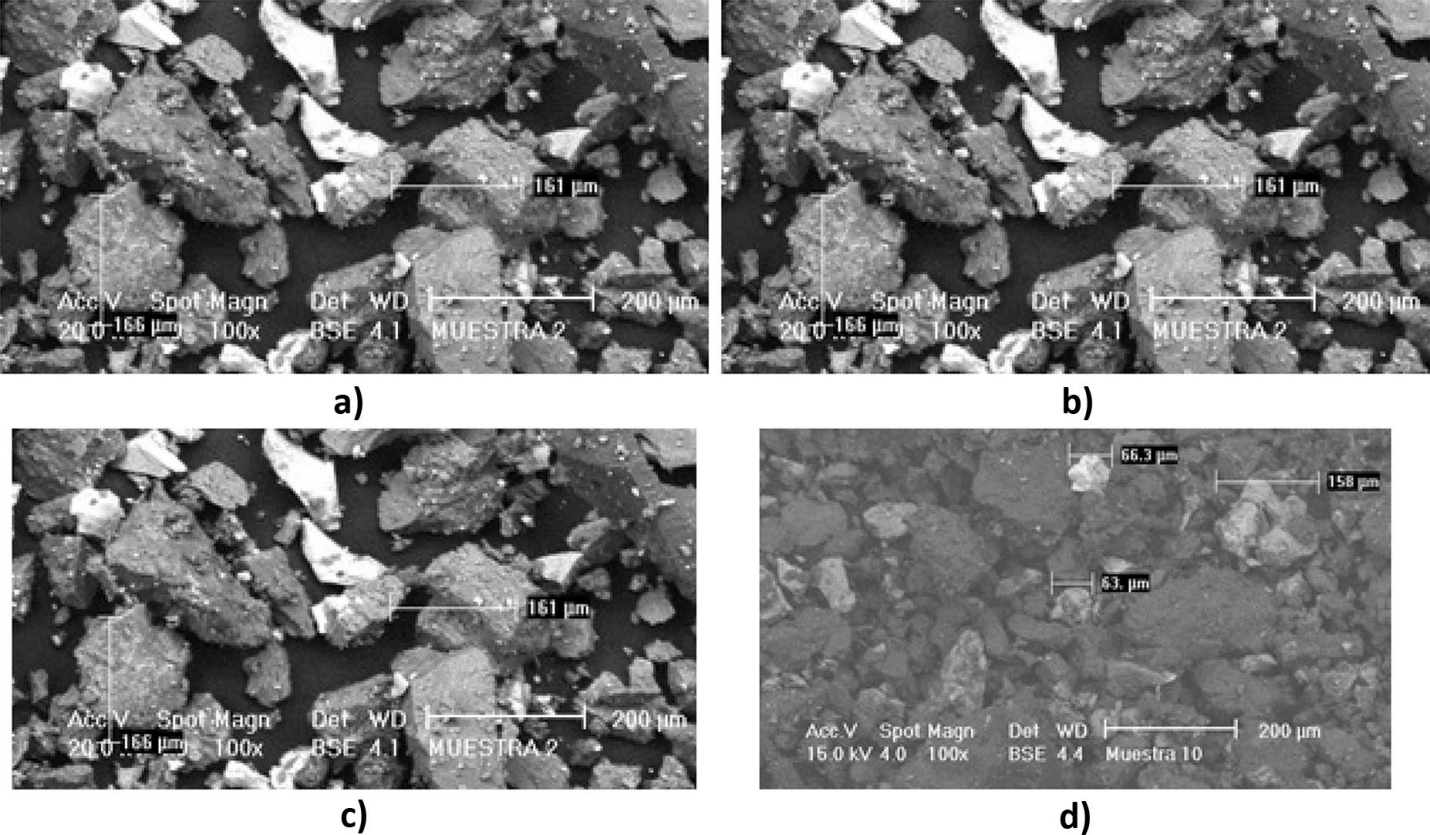
Different samples visualized and measured at × 100 under the scanning electron microscope (SEM). **a** z-2, **b** z-3, **c** z-9, and (**d**) z-10

The minimum opening in sieving by the mechanical method was 37 microns, finding that the largest particle size was 0.92 mm (Fig. 1). As shown in Fig. 1, between 0.5 and 1% of the samples have a size smaller than 37 microns. Given the very small measurement of the particles, size was determined using the scanning electron microscope (SEM) (Fig. 2). Due to the above, it is very likely that the particles enter into the body. Those that are smaller than 100 microns form aerosols when they are suspended in the air and can be inhaled. Given the particle size, all the samples can form aerosols. The sample z-3 which has the highest probability of forming it because 69% of its composition has a smaller size than 100 microns (Gavilán-García et al., 2020). In addition, when they measure less than 10 microns, they can be accumulated in the respiratory system and cause physical damage if they are insoluble and toxicity if they are solubilized, especially breathable particles (< 4 microns) which can pass into the bloodstream (Gavilán-García et al., 2020; Puga et al., 2006).

As we can see in Fig. 2, the different shades of gray can be observed, which indicates different densities between the particles for which, a qualitative chemical analysis was carried out. Making a spot in different areas of the samples (Table 2) and finding that the dark parts are made up of silicates (SiO_4_) and carbonates (CO_3_) which are the least dense. The white parts are made up mainly of pyrites with higher density. The existence of these phases was confirmed using the FTIR-ATR technique.

**Table 2:**
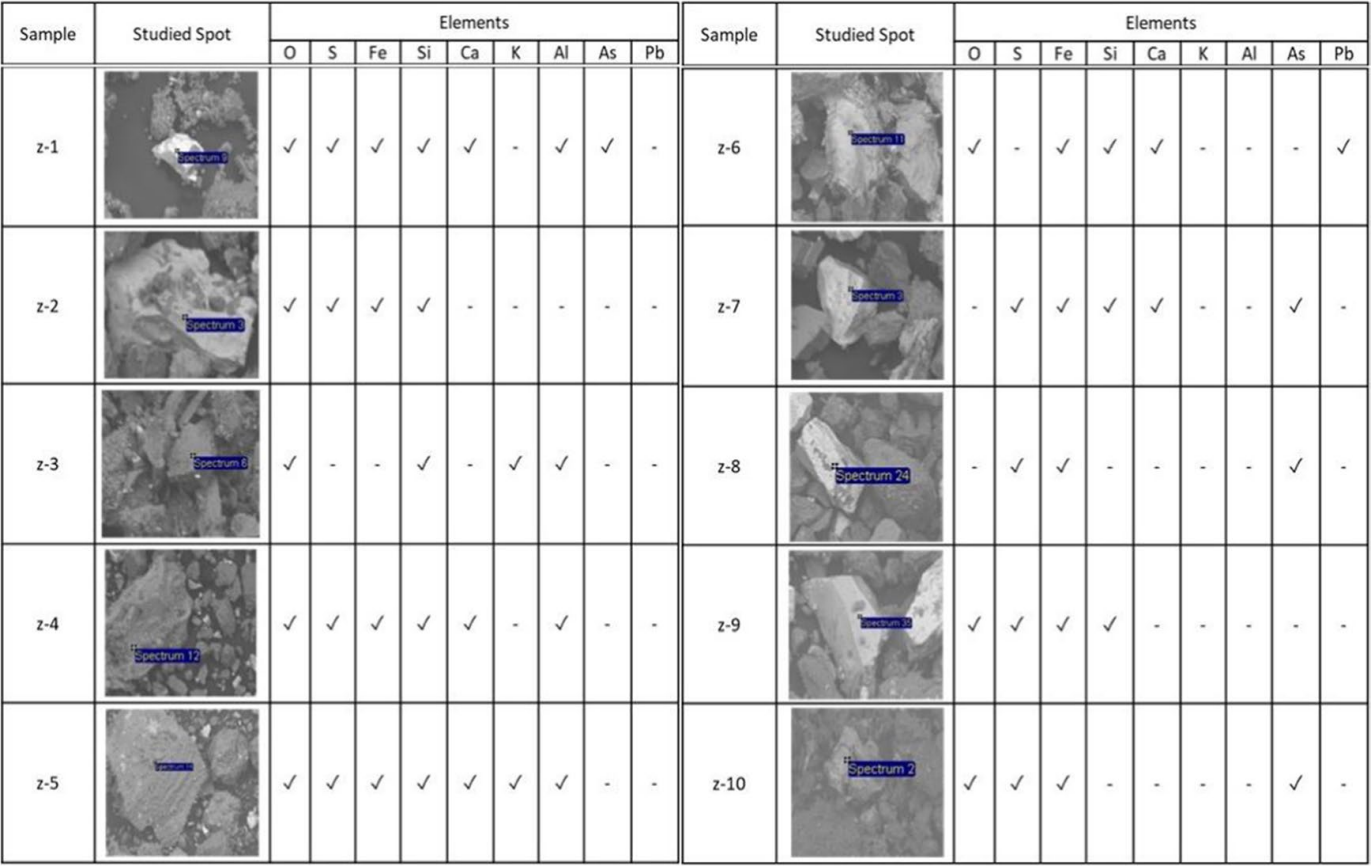
Elements identified by point elemental analysis

According to the Tables 2 and 3, there are potential toxic elements such as arsenic and lead present in the samples. Furthermore, in some samples where sulfur was detected, however, analyzing the contrast of the images, it is noted that the surrounding particles that contain this element are mostly carbonates (calcite, magnesite, smithsonite, principally), so in the presence of water, they can neutralize to sulfuric acid generated by sulfur and thus avoiding the formation of acid drainage and the leaching of toxic metals, which is also justified with the pH values obtained (García et al., 2017; Salas Urviola et al., 2020a, 2020b).

**Table 3:**
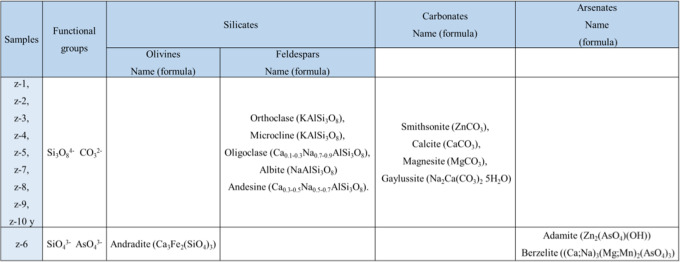
Mineralogical compositions of tailings by FTIR-ATR

**Fig. 3:**
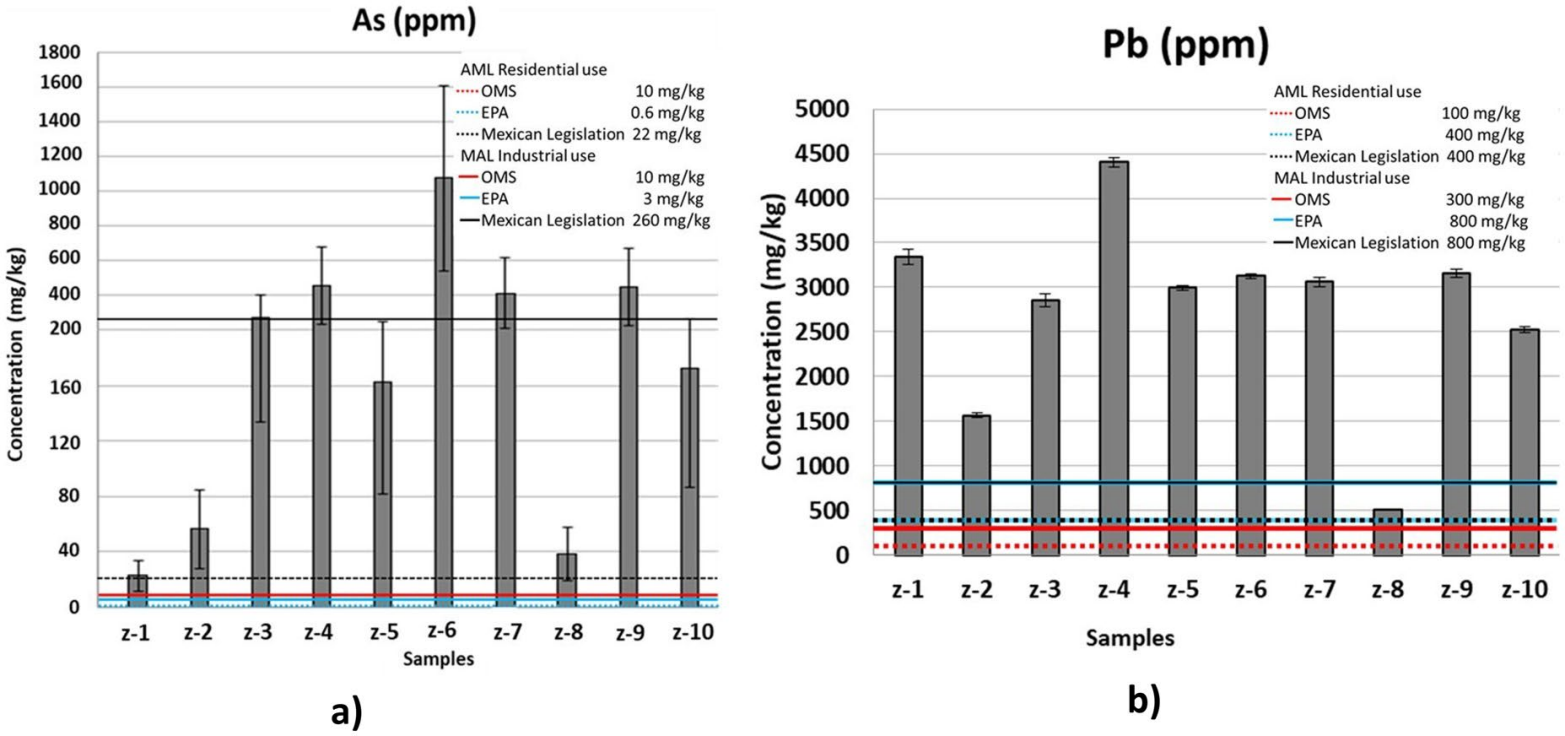
Total concentration of hazardous toxic elements from the acid digestion of the samples, and their allowable maximum limit (AML) according to WHO, EPA, and Mexican Legislation (NOM-147-SEMARNAT/SSA1-2004) standard: **a** As and (**b**) Pb

According to various standards (World Health Organization, WHO; Environment Protection Agency, EPA and Mexican legislation (NOM-147-SEMARNAT/SSA1-2004))(Semarnat, 2007; US EPA, 2022; Guidelines for drinking-water quality, 2022), considered for residential land use, the concentration of As and Pb in all samples is higher than the allowable maximum limit (AML) (Fig. 3). For industrial land use, the AML of WHO and EPA legislation for As is exceeded, while for Mexican legislation with a value of 260 ppm, some samples exceeded the limit, except for z-1, z-2, z-5, z-8, and z-10. All samples considered for industrial land use exceed the AML established by the three different legislations for Pb, except for sample z-8, which meets the limits established by EPA and Mexican legislation. The literature suggests that due to the measured particle size, both As and Pb can cause harm to the population and the environment through direct inhalation of the tailings. However, the measured pH values and sample characterization suggest that the abundance of carbonates and the basic pH of the samples will result in low dilution of both As and Pb in bodies of water, forming insoluble species and not generating acid drainage (Armienta et al., 2016; Gavilán-García et al., 2020; Gavilán García et al., 2017 et al., 2017; Kossoff et al., 2014; Salas Urviola et al., 2020a, 2020b).

### AERMOD modeling

WRF and MMIF coupled data generated a single grid cell (12 km) representative of the entire domain. Modeling took into account each hour and variable in a whole year (2018). The generated surface and vertical meteorology files were input into AERMOD to estimate the dispersion of air pollutant emissions from mining tailings. With the application of the AERMOD model, the distribution of PM_10_ (plume forecast) over the urban area was obtained (Jayadipraja et al., 2020).

The digital elevation model is represented in Figure S6, Supplementary Materials, and the mesh was used to calculate the number of receptors considering a spacing of 0.5 km. The final number of receptors in the study area was 10,201. With the help of the AERMAP module, the images were generated.

**Fig. 4:**
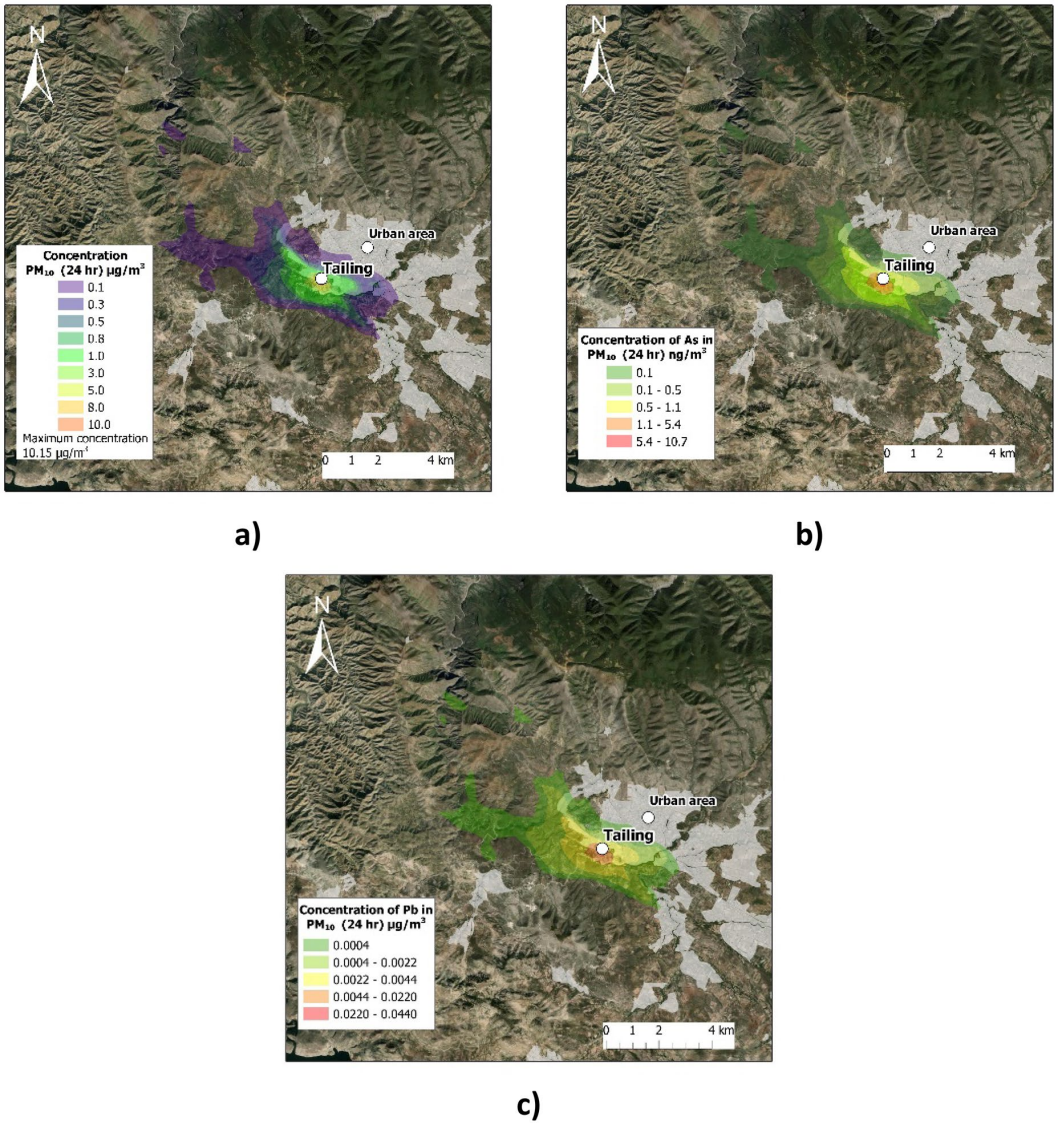
Worst case atmospheric scenario for PM_10_ (24 – hour mean), the maximum concentration is located close to the emission source. **a** Dispersion plume of PM10. **b** Estimate of the dispersion plume of As. **c** Estimate of the dispersion plume of Pb

According to the emission factors, the WRF meteorology, and the 365-day modeling for the year 2018; the estimated impact on air quality by PM_10_, Pb, and As was generated for the study area. These values are shown in Table 4. Pb and As concentrations were estimated based on the highest concentrations measured in samples from mining tailings. Regarding the maximum permissible limits allowed for Pb and As concentrations in PM_10_ particles, AML was searched both in the Mexican regulations, as well as in the USEPA and in the World Health Organization (WHO), and no limits for As were found. For this reason, it was decided to use the values established in the European Union. The calculated values are compared with the thresholds recommended by Europe Union Air Quality Standards (EU AQS). Figure 4 shows the concentrations for the worst modeled scenario, the maximum average concentration (24-h) of PM_10_ is close to the study area.

**Table 4:** Emissions and impact on air quality for PM_10_ related to mining processes by study area

Process	Emissions (g/seg m^2^)	Impact to air quality^1^		
	PM_10_	PM_10_ (µg/m^3^)	Pb (µg/m^3^)	As (ng/m^3^)
Wind erosion (exposed areas)	6.754 E − 07	10.15	0.04	10.90
EU AQS		50.00	0.50^2^	6.00^2^
Percentage (%)		− 79.70	− 92.00	+ 81.63

highest contribution estimated considering the maximum concentration of the samples

^1^Maximum (24 h)

^2^The standard mentions that an average of 1 year should be considered; however, the maximum daily concentration calculated in the model was taken as a reference

Considering the direction and speed of the wind as well as the concentrations of the particles, the dispersion cloud travels to the opposite side of the highest concentration of human settlements. However, there is an impact on the population. According to the estimation of the concentration of the particles from the dispersion model, it was calculated that this site could contribute up to 10.15 μg/m^3^ of particles to the air quality of the zone.

Other emission sources are not considered; however, in the study area, other companies are engaged in mining, generating tailings exposed to dispersion.

Since the study area is characterized by its mining activity and the wind direction coming from the northeast, the particles generated by the different activities and mining processes can be dispersed and deposited in human settlements.

The study area does not have an air quality monitoring system; however, monitoring and measurements of TSP, PM_2.5_, and PM_10_ concentrations in the air have been carried out. For PM_10_, 70 samples were measured and collected at five sites during February–March 2017. The range of measurements for PM_10_ was 23.6 to 80.1 µg/m^3^ (Corona Sánchez et al., 2021). This data suggests that if we take the 99th percentile (5.36 µg/m^3^) concentration of the study area and compare it with the maximum value monitored (80.1 µg/m^3^), it contributes 6.69% to the air quality levels at the site.

According to the results of the meteorological modeling with WRF, the AERMOD air quality modeling system (365 days of the year 2018), and the emission factors of particles (wind erosion), it is estimated that the study area (mining activity) generates an impact on air quality due to the dispersion of PM_10_ with airborne concentrations of 0.28 µg/m^3^ annual average (24 h), 5.36 µg/m^3^ for the 99th percentile (24 h) and 10.15 µg/m^3^ the maximum concentration (24 h). Compared to the Mexican Standard Levels, it represents 0.70, 7.15, and 10.15% of the threshold, respectively.

It is essential to mention the practical implications and difficulties of air quality modeling (Huertas et al., 2012), for example, when there is no air quality monitoring system.

## Conclusions

This research evaluated the exposure to the population and the danger of tailings belonging to an active mine in Mexico through the characterization of its tailings and using a dispersion model. The modeling results estimated that the dispersion of particles from the tailings dam can contribute up to 10.15 µg/m^3^ of PM_10_ to the air quality of the site, which, according to the characterization of the samples obtained, it could be dangerous for human health and due to estimated concentration superior of 0.04 µg/m^3^ of Pb and 10.90 ng/m^3^ of As. In this way it is recommended that different treatments be applied to the tailings in order to mitigate potential emissions.

It is suggested that in the urban area, the installation of an air quality monitoring station be promoted, specifically for particles (PM_10_ and PM_2.5_). In addition, it is recommended to carry out particle monitoring for at least 1 year in the study area to analyze the concentrations of these atmospheric pollutants and take biological samples to analyze the concentrations of Pb and As.

## Supplementary Information

Below is the link to the electronic supplementary material.

Supplementary file1 (DOCX 524 KB)

## Data Availability

The datasets generated during and/or analyzed during the current study are available from the corresponding author on reasonable request.
