# Supplementary material for: Evaluation of the danger of a tailings pile belonging to an active mine through its characterization and a dispersion model
Source: Environ Monit Assess. 2023 Jun 26;195(7):889. doi: 10.1007/s10661-023-11475-4 (PMC10293379; doi:10.1007/s10661-023-11475-4)
Supplement: Supplementary file 1 — Supplementary file1 (DOCX 524 KB) [file 10661_2023_11475_MOESM1_ESM.docx]

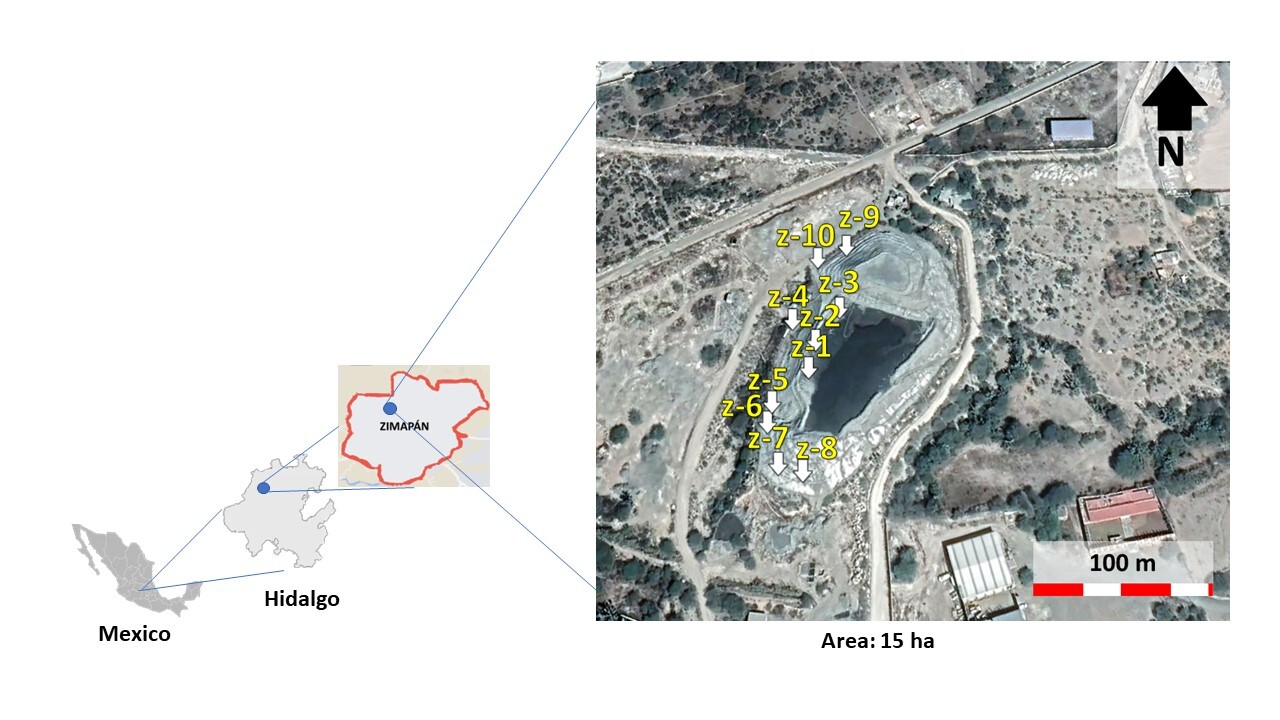


**Figure S1.** Localization of site and sampling points into tailing dam.

**
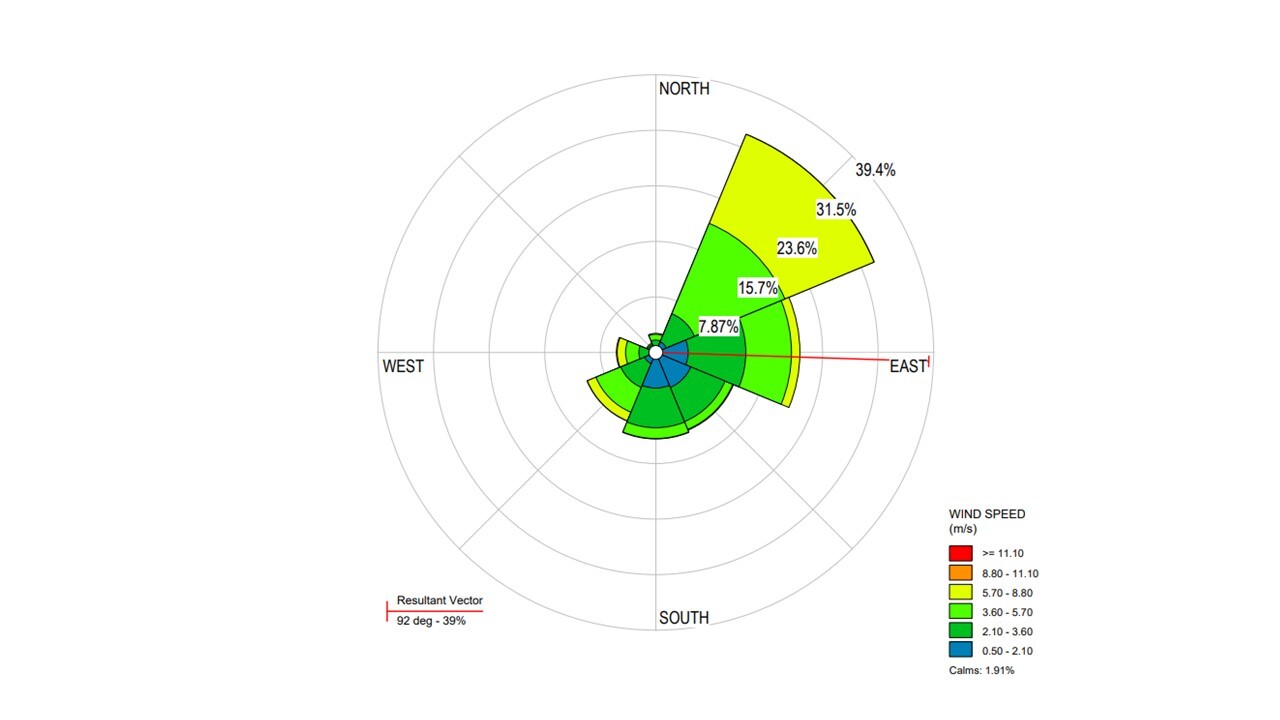
**

**Figure S2.** Wind rose of the study area according to WRF model in 2018


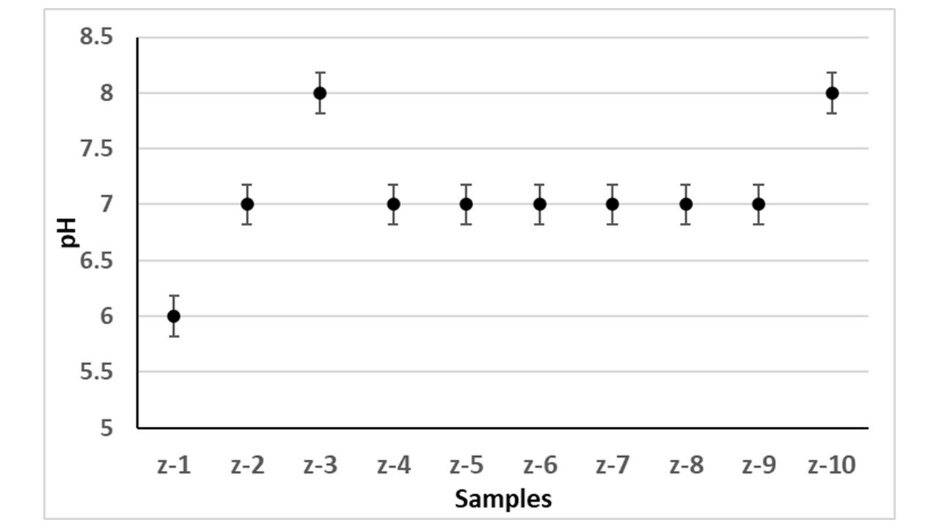


**Figure S3.** pH of different samples.


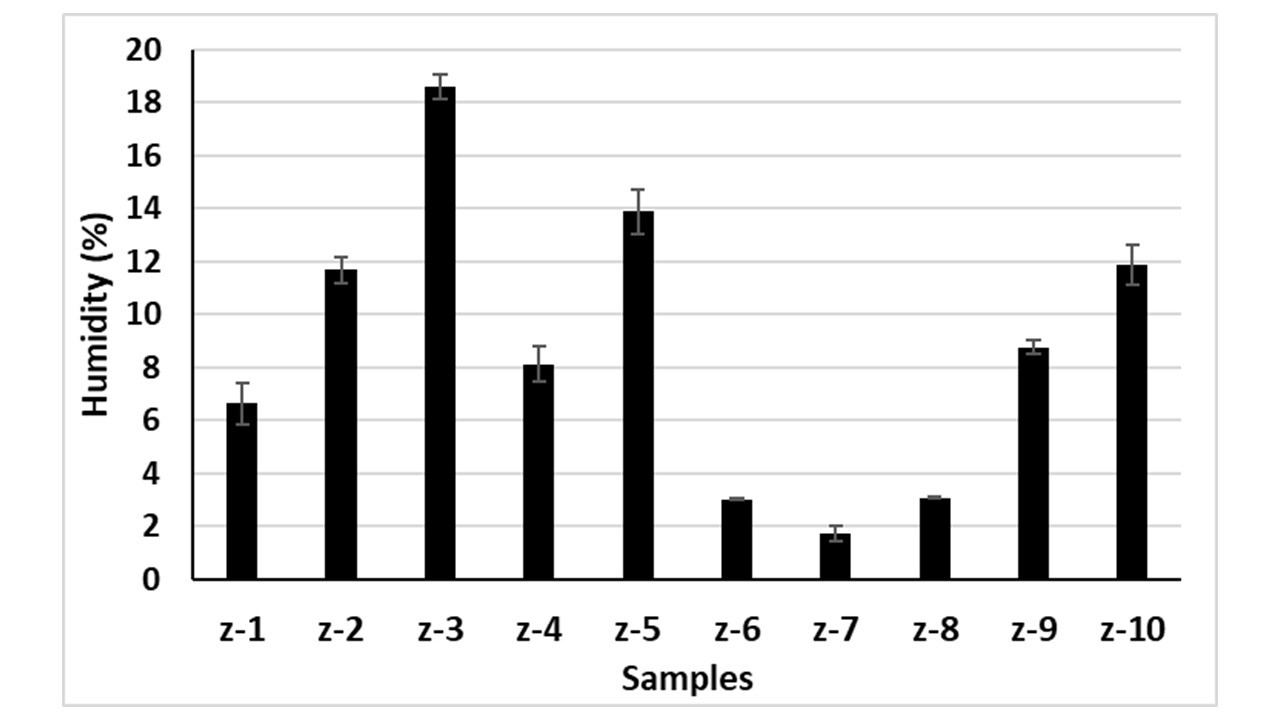


**Figure S4.** Percentage of humidity of different samples.

| **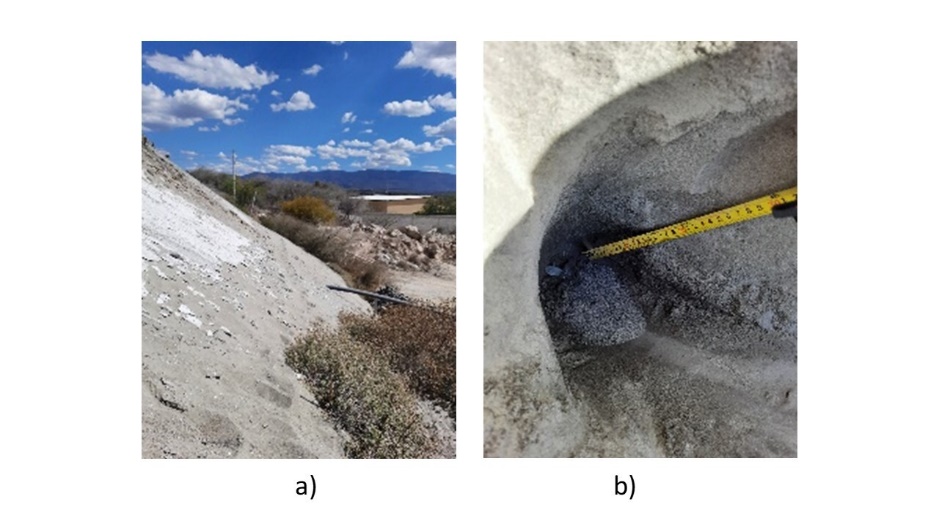** | **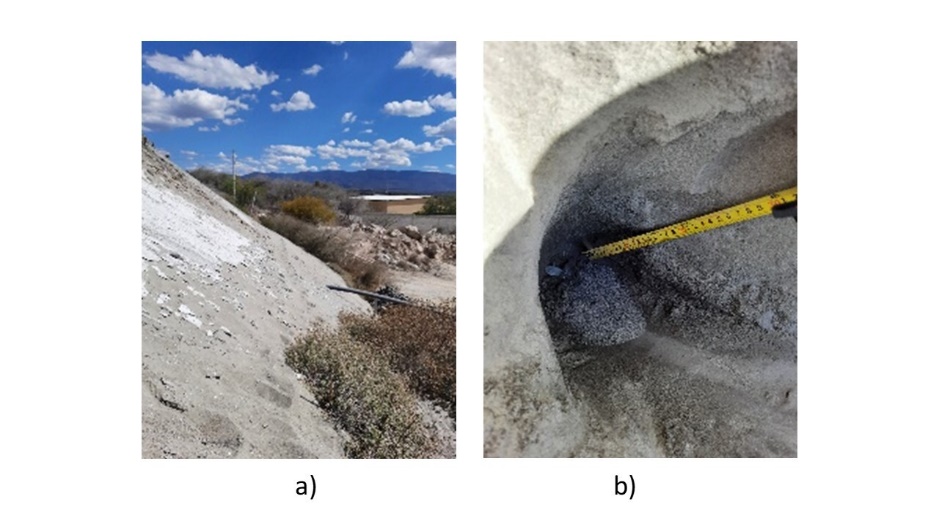** |
| --- | --- |

**Figure S5.** The way in which the tailings are found at the disposal site.


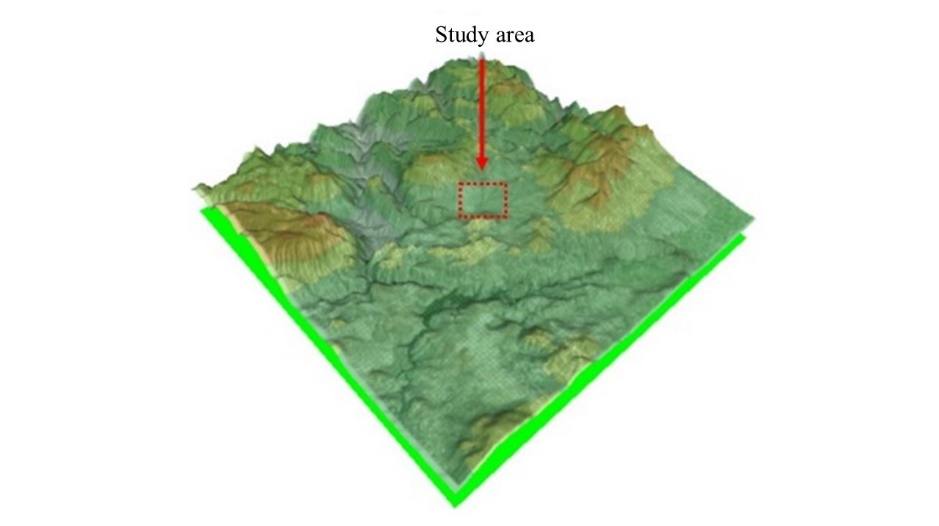


**Figure S6.** Terrain modeling.
